# Case Report: A rare cause of acute abdomen: retrograde efferent loop intussusception into the gastric remnant following Billroth II gastrectomy

**DOI:** 10.3389/fmed.2026.1814401

**Published:** 2026-03-31

**Authors:** Li-Hao Deng, Zheng-Hong Jiang, Li-bin Deng

**Affiliations:** Department of Gastrointestinal, Hernia and Abdominal Wall Surgery, Affiliated Hospital of Chengdu University, Chengdu, Sichuan, China

**Keywords:** Billroth II gastrectomy, distal gastrectomy, gastric remnant, gastrotomy, retrograde intussusception

## Abstract

This paper reports a special case in which retrograde intussusception of the efferent loop through the gastrojejunostomy into the gastric remnant led to closed-loop intestinal obstruction and small bowel ischemia. The patient was a 69-year-old male with a history of subtotal gastrectomy, who was admitted for acute abdominal pain. Initial computed tomography (CT) findings were inconclusive, while contrast-enhanced CT suggested the possibility of intussusception. Emergency surgery confirmed the diagnosis, and the patient underwent gastrotomy with reduction, followed by partial small bowel resection and anastomosis. The postoperative recovery was uneventful. This case highlights the rare location, diagnostic challenges, and the importance of multidisciplinary management in postgastrectomy internal hernia/intussusception. Based on a literature review, we provide an in-depth discussion of its pathogenesis, imaging characteristics, and surgical management strategies.

## Introduction

Long-term complications may arise after subtotal gastrectomy, particularly following Billroth II or gastrojejunostomy reconstruction, with internal hernias (e.g., Petersen's hernia) representing one of the critical causes of acute intestinal obstruction. However, retrograde intussusception of the afferent or efferent limb into the gastric remnant lumen through the gastrojejunal anastomosis constitutes an extremely rare acute abdominal emergency. Its clinical presentation is non-specific, radiographic diagnosis challenging, often leading to delayed treatment and high mortality. This article aims, through a typical case presentation and literature review, to summarize the clinical characteristics, diagnostic key points, and therapeutic principles of this condition, thereby enhancing clinicians' awareness of this rare yet life-threatening complication.

## Case presentation

A 69-year-old male presented to the emergency department with a 6-h history of sudden-onset severe epigastric colicky pain accompanied by nausea and vomiting of gastric contents. His past medical history was significant for distal gastrectomy performed approximately 38 years earlier for gastric ulcer. According to the original surgical records, the procedure involved resection of approximately 50% of the distal stomach, including the pylorus, antrum, and part of the gastric body. Reconstruction was performed using an antecolic Billroth II gastrojejunostomy, in which the gastric remnant was anastomosed end-to-side to the jejunum approximately 20 cm distal to the ligament of Treitz, resulting in an efferent limb length of approximately 20 cm. Of note, the original surgical record did not mention whether the mesenteric defect was closed at the time of the initial operation.

Upon admission, the patient's BMI was 18.5 kg/m^2^. Laboratory findings revealed: hemoglobin 114 g/L, neutrophil count 6.5 × 10^9^/L (83.1%), and serum sodium 132.3 mmol/L.

**Figure 1:**
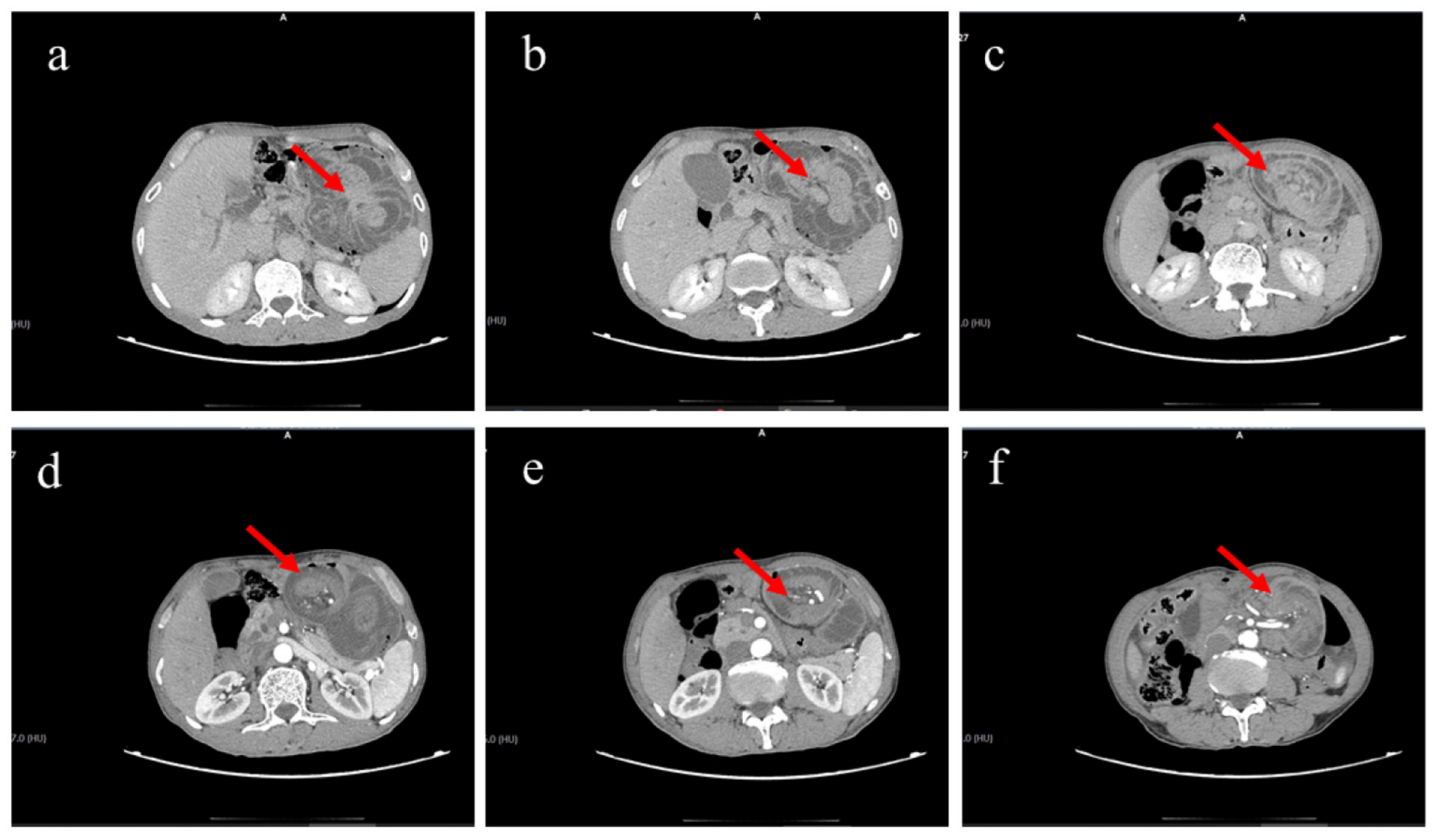
Emergency abdominal CT findings. **(a–c)** Non-contrast CT images showing thickened small bowel wall in the left upper quadrant (arrows), with partial herniation of small bowel into the gastric lumen and intussusception. **(d–f)** Contrast-enhanced CT images revealing small bowel volvulus in the left upper quadrant and gastrojejunal intussusception (arrows).

Initial emergency abdominal CT suggested partial small bowel obstruction in the abdominopelvic cavity. A thick-walled tubular structure was observed in the left upper quadrant, located within or closely related to the gastric lumen, the nature of which remained indeterminate (Figures 1a–c). Differential diagnoses at that time included gastric content retention, partial gastric intussusception, or internal herniation of the small bowel.

The patient was initially managed conservatively with fasting, continuous gastrointestinal decompression, intravenous fluids (5% glucose normal saline supplemented with 4 g of 10% sodium chloride to correct hyponatremia), antibiotic therapy (cefmetazole 2 g intravenously every 12 h), and gastric protection (omeprazole 40 mg intravenously once daily). However, conservative management proved ineffective, prompting an enhanced abdominal CT scan for further evaluation.

As symptoms progressively worsened, contrast-enhanced abdominal CT was performed. The findings indicated structural derangement of the small intestine and gastric lumen in the left lower abdomen, raising high suspicion for small bowel volvulus with gastrointestinal intussusception (Figures 1d–f). Internal herniation (e.g., Petersen's hernia) could not be excluded. The presentation was accompanied by partial intestinal obstruction. Given the potential risk of intestinal ischemia, emergency laparoscopic exploration was decided upon.

**Figure 2:**
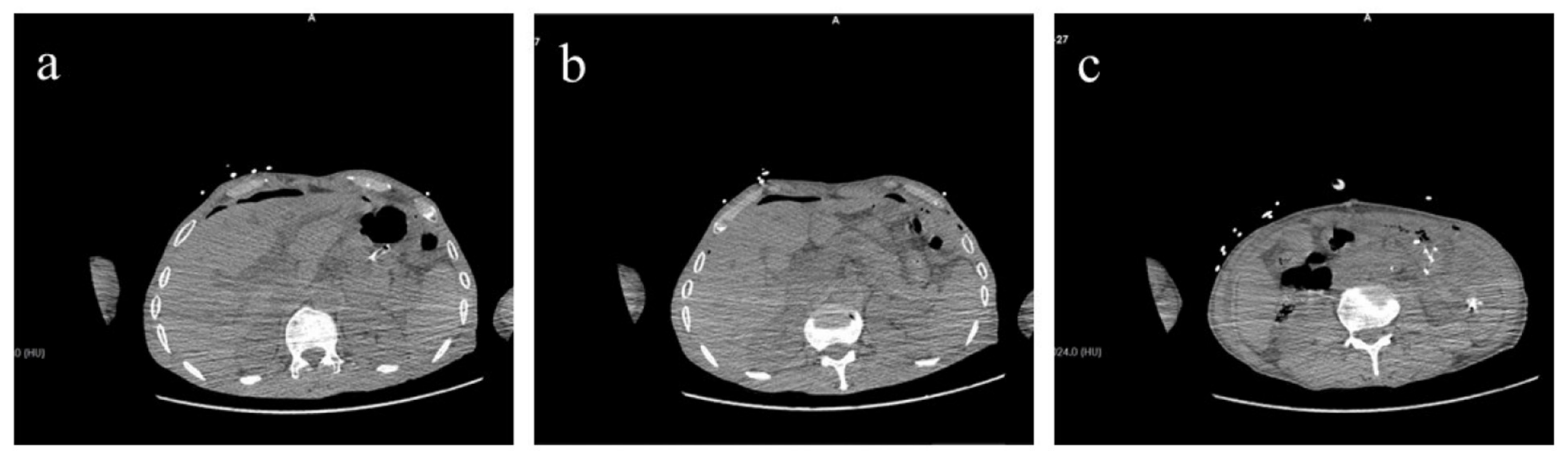
Postoperative imaging confirming resolution of obstruction. **(a–c)** Sequential axial CT slices demonstrate absence of small bowel air-fluid levels and normalization of the intestinal lumen, confirming successful surgical management of the obstruction.

**Figure 3:**
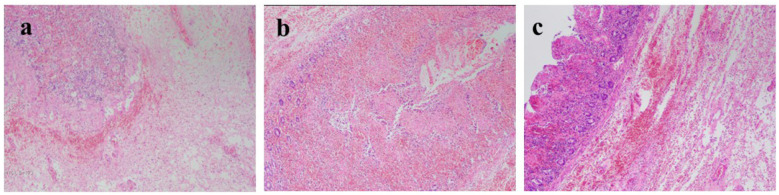
Microscopic findings of the resected bowel. **(a–c)** Images show transmural inflammatory infiltrate, extensive hemorrhage, and mucosal necrosis with sloughing, consistent with a severe, non-specific inflammatory process.

**Laparoscopic Exploration:** Intra-abdominal adhesions were observed, with the intussuscepted bowel segment clustered in the left upper quadrant corresponding to the gastric remnant region. Due to the substantial volume of intussuscepted bowel and the difficulty of reduction under laparoscopy, conversion to open laparotomy was performed. **Open Exploration:** adhesions between the stomach, diaphragm, and spleen were lysed, and the gastric remnant was exteriorized. It was clearly identified that the small bowel had intussuscepted from the efferent limb and further herniated retrogradely through the gastrojejunal anastomosis into the lumen of the gastric remnant. A significant length of small bowel was palpable within the gastric lumen, consistent with a closed-loop obstruction. Direct manual reduction in the extracorporeal field was unsuccessful. Due to pronounced bowel wall edema, forceful traction was avoided to prevent iatrogenic bowel injury; therefore, an anterior gastrotomy was performed. Upon entering the gastric lumen, a substantial amount of hemorrhagic fluid was observed, and multiple segments of the herniated bowel appeared dusky and ischemic. Following gastrotomy, the hernial ring was relaxed, and the intussuscepted small bowel was gradually reduced by gentle manual pressure at the gastrojejunal anastomosis site. After confirming the integrity of the gastric remnant and anastomosis with no active bleeding, the gastrotomy incision was closed using a barbed continuous suture. Following reduction, bowel viability was assessed. Approximately 60 cm of the small bowel segment was found to be irreversibly ischemic and was therefore resected, with a subsequent side-to-side anastomosis performed. To prevent recurrence, the necrotic efferent loop was resected, and both efferent loop fixation and closure of the mesenteric defect were performed. The patient was transferred to the Intensive Care Unit (ICU) postoperatively. The patient's postoperative management included a multifaceted regimen comprising broad-spectrum antibiotics for infection prophylaxis, acid-suppressive therapy for stress ulcer prevention, and total parenteral nutrition (TPN). Specific replacement therapy with albumin and fibrinogen was administered to correct hypoalbuminemia and coagulopathy. Concomitantly, non-invasive ventilation (NIV), prophylactic anticoagulation, and early mobilization were instituted. The patient's postoperative course was uneventful; a follow-up abdominal CT scan confirmed resolution of the obstruction (Figure 2), and histopathological examination of the surgical specimen revealed inflammatory changes in the intestinal wall (Figure 3), thereby establishing the definitive diagnosis. He made a satisfactory recovery and was discharged on postoperative day 14.

The timeline illustrates the key chronological events from the initial gastrectomy in 1986 to the final discharge in August 2025. The patient presented with acute abdominal symptoms on July 26, 2025, and failed conservative management. Emergency CT confirmed small bowel volvulus with intussusception. On July 27, 2025, the patient underwent emergency laparoscopic converted to open surgery, including adhesiolysis, manual reduction of intussusception, and resection of approximately 60 cm of ischemic small bowel with side-to-side anastomosis. Postoperative intensive care was required, and the patient was discharged uneventfully 14 days after admission.

## Discussion

**Figure 4:**
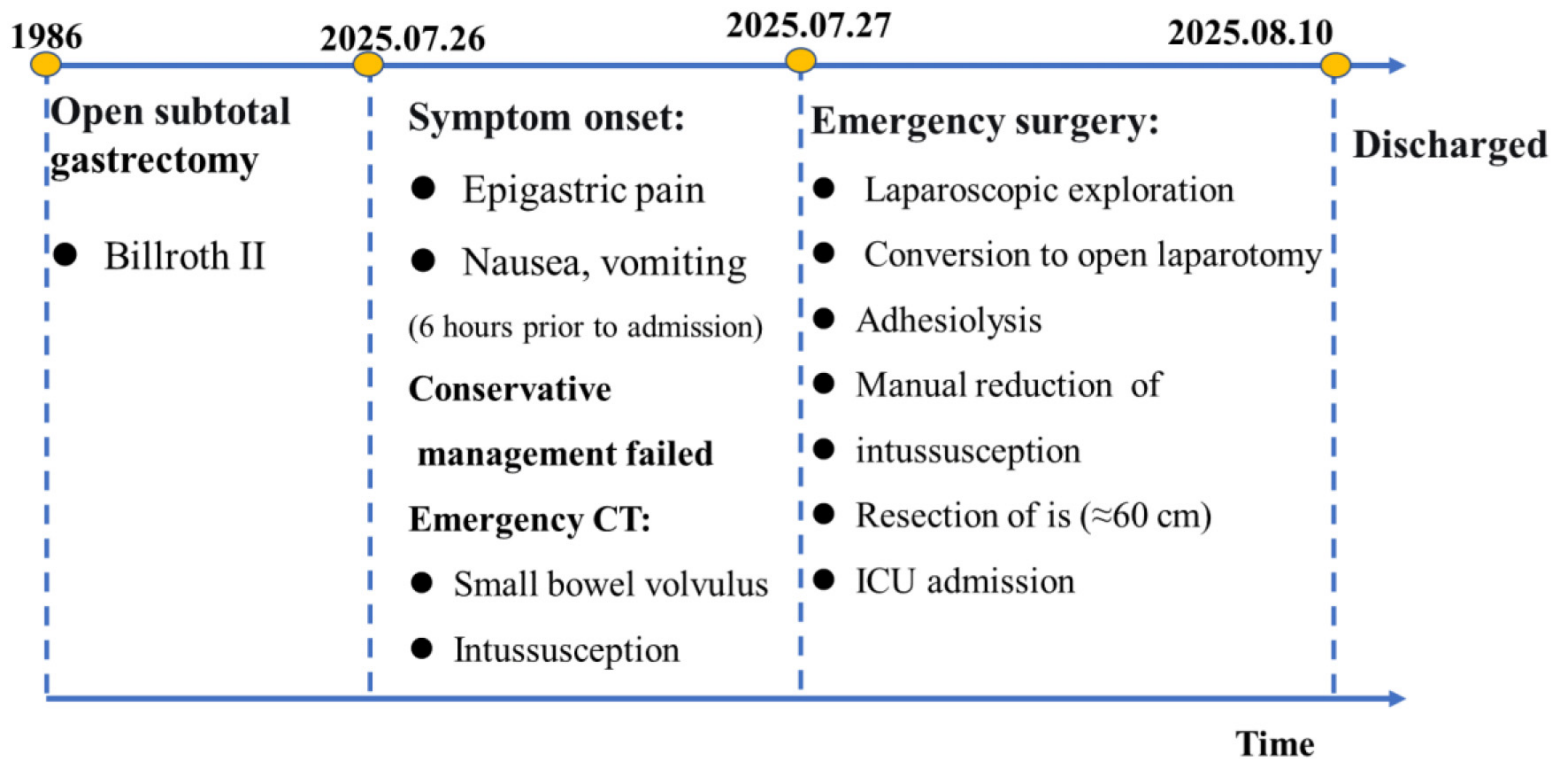
Clinical timeline of the patient's presentation, diagnosis, surgical management, and recovery.

This report describes a rare case of retrograde efferent loop intussusception through the gastrojejunal anastomosis into the gastric remnant, occurring nearly four decades after distal gastrectomy with Billroth II reconstruction. The patient presented with acute abdominal pain and vomiting, and initial conservative management failed to alleviate symptoms. Emergency contrast-enhanced CT raised suspicion of small bowel volvulus with intussusception, prompting urgent surgical exploration. Intraoperative findings confirmed retrograde herniation of the efferent limb into the gastric remnant, leading to closed-loop obstruction and segmental small bowel ischemia. Surgical management included reduction via gastrotomy, resection of approximately 60 cm of non-viable small bowel with primary anastomosis, and preventive measures including efferent loop fixation and mesenteric defect closure. The patient made an uneventful recovery and was discharged 14 days postoperatively (Figure 4). At the 6-month follow-up, the patient remained asymptomatic with no evidence of recurrence on imaging. This case highlights the diagnostic challenges and the importance of timely surgical intervention in this rare but life-threatening complication following gastrectomy.

Retrograde efferent loop intussusception through the anastomotic stoma into the gastric remnant is an extremely rare yet life-threatening complication following subtotal gastrectomy, representing a special type of internal hernia. Early diagnosis and appropriate treatment are crucial for prognosis; delayed diagnosis is associated with postoperative complication rates as high as 50% and mortality reaching 25% (1), even with surgical intervention.

The pathogenesis of this condition is multifactorial, involving both anatomical predispositions and functional disturbances. Anatomical factors contributing to this complication include an excessively long efferent limb, excessive tissue inversion at the anastomotic site, postoperative adhesions (2), anastomotic stenosis, thinning or elongation of the mesentery, and sudden increases in intra-abdominal pressure. These anatomical variations may compromise gastrointestinal motility and predispose the patient to intestinal spasm and abnormal peristalsis (3). Specifically, an excessively long efferent loop and mesentery increase intestinal mobility and may lead to enlargement of the mesenteric hiatus, thereby predisposing to internal hernia formation. Functional disturbances play an equally important role (1, 4). One proposed mechanism is intestinal pacemaker disruption. During Roux-en-Y reconstruction, transection of the jejunum separates the distal limb from the proximal jejunal pacemaker, resulting in decreased pacemaker potential distal to the anastomosis and activation of ectopic pacemakers in the distal jejunum. These ectopic pacemakers generate aberrant pacing potentials that propagate distally, leading to delayed and stagnant emptying near the anastomosis, which may predispose to intussusception (5).

Additional functional factors include altered intestinal motility secondary to various perioperative insults, such as intestinal ischemia, bacterial translocation-induced intra-abdominal inflammation, extensive intraoperative manipulation resulting in bowel wall edema and serosal injury, extensive preperitoneal dissection, electrolyte imbalances, local hypoxia, and the effects of anesthetic and postoperative medications (6–8). All of these factors may contribute to the disruption of coordinated intestinal peristalsis.

Risk Factors and Predisposing Conditions, A decrease in body mass index (BMI) following gastrectomy may be a potential risk factor, possibly by increasing mesenteric defects and elevating the risk of herniation. Gender may also play a role, potentially related to differences in body fat distribution between sexes (9). In males, body fat is predominantly distributed within the abdominal cavity; following gastrectomy, the reduction of mesenteric fat leads to a more pronounced increase in mesenteric space (2, 10). Additionally, subtotal gastrectomy involving resection of the gastric wall and greater omentum results in increased free space in the upper abdomen, leading to greater mobility of the small intestine and thereby elevating the risk of internal hernia formation (11). Notably, in the context of bariatric surgery, patients undergoing Roux-en-Y gastric bypass face an additional risk factor: rapid weight loss-induced mesenteric relaxation (12–15). The resultant increase in intestinal mobility and redundancy further predisposes to intussusception.

Surgical Prevention, given these mechanisms, closure of the mesenteric hiatus has been shown to significantly reduce the incidence of internal hernia (16, 17). This preventive measure should be considered during the initial gastric resection, particularly in patients with identifiable risk factors.

Correlation with the Present Case, in the present case, the patient exhibited several of these predisposing factors. He underwent distal gastrectomy approximately 38 years ago, with resection of approximately 50% of the distal stomach, including the pylorus, antrum, and part of the gastric body. Reconstruction was performed using an antecolic Billroth II gastrojejunostomy, in which the gastric remnant was anastomosed end-to-side to the jejunum approximately 20 cm distal to the ligament of Treitz, resulting in an efferent limb length of approximately 20 cm. Of note, the original surgical record did not mention whether the mesenteric defect was closed at the time of the initial operation. Additionally, the patient had a low BMI (18.5 kg/m^2^) with associated mesenteric thinning. The combination of a long efferent limb, potential unclosed mesenteric defect, low BMI, and male sex—with its characteristic fat distribution—likely contributed to the development of retrograde efferent loop intussusception three decades after the initial surgery. This case underscores the importance of considering late-onset internal hernia in patients with remote history of gastrectomy presenting with acute abdomen.

The diagnosis of internal hernia remains challenging due to its non-specific clinical presentation (18). Most patients present with vague symptoms such as epigastric pain, nausea, and vomiting. Furthermore, internal hernia may undergo spontaneous reduction. When complicated by intestinal obstruction, delayed recognition can lead to severe consequences, including strangulation, necrosis, and perforation. Contrast-enhanced abdominal CT has emerged as the modality of choice for diagnosing internal hernia, with reported preoperative diagnostic accuracy of 80%−90% in patients with a history of gastrectomy. The presence of mesenteric swirl sign on CT in a patient with prior gastric surgery and acute abdominal pain should raise high suspicion for internal hernia, prompting early surgical intervention to prevent intestinal strangulation (19). However, a negative CT finding does not definitively exclude internal hernia; diagnostic laparoscopy may be considered in symptomatic patients with pertinent surgical history (18, 20).

Retrograde efferent loop intussusception into the gastric remnant represents an extremely rare but life-threatening subtype of internal hernia following subtotal gastrectomy. Its diagnosis relies on clinical suspicion based on remote surgical history and meticulous interpretation of high-resolution CT imaging. The cornerstone of management is timely surgical intervention, encompassing flexible application of gastrotomy for safe reduction, definitive resection of ischemic bowel segments when necessary, and comprehensive perioperative care. This case provides valuable clinical experience for managing this complex surgical emergency.

As reported in previous cases, in the common variant, patients may present with acute, subacute, or intermittent symptoms (often due to spontaneous reduction of the intussusception). Manifestations typically include abdominal pain and distension of a relatively mild nature, with a gradual or progressive onset. These cases often respond to conservative management. In advanced stages where bowel necrosis develops, peritoneal signs may eventually appear (21–23). Computed tomography (CT) findings in this variant generally show dilated bowel loops and air-fluid levels, without the characteristic “whirl sign” indicative of torsion (24).

In contrast, the present case represents a fulminant variant. The patient presented with acute, severe colicky abdominal pain, progressing rapidly to overt peritonitis and signs of hemodynamic instability/shock. The onset was sudden, with an extremely rapid progression. Early-stage peritoneal signs were evident due to swift intestinal ischemia. CT imaging revealed a distinct “whirl sign” indicating mesenteric torsion, with clear invagination of the small bowel into the gastric remnant. Intraoperative exploration confirmed the presence of small bowel necrosis. This pattern necessitates emergency surgical intervention, as delayed management carries a high risk of mortality.

## Data Availability

The original contributions presented in the study are included in the article/supplementary material, further inquiries can be directed to the corresponding authors.
